# Elective Endovascular Treatment of Unruptured Intracranial Aneurysms

**DOI:** 10.7759/cureus.27515

**Published:** 2022-07-31

**Authors:** Evan M Krueger, Hamad Farhat

**Affiliations:** 1 Neurosurgery, Advocate Aurora Health, Downers Grove, USA

**Keywords:** neurocritical care unit, elective, procedural complications, aneurysm, endovascular

## Abstract

Background

Elective endovascular treatment (EVT) of unruptured intracranial aneurysms (UIA) is a commonly used treatment modality. However, the appropriate post-procedure management is not well-defined.

Methods

This was a single-center, retrospective review of all adults undergoing EVT of UIA performed between January 1, 2010, and March 31, 2020. Patients with any current intracranial hemorrhage or clinical symptoms severe enough to warrant emergent intervention were excluded.

Results

Sixty-seven UIA were treated on 58 patients. The mean dome diameter was 6.6 mm (2-20, ±3.9), the most common parent vessel was the internal carotid artery (43.2%, 29/67), and sole flow diverter stents were the most common device used (46.2%, 31/67). Post-treatment, 43.2% (29/67) patients went to the neurocritical care unit (NCCU). The mean NCCU length of stay (LOS) was 1.07 days (range 1-4, ±0.5), and 96.6% (28/29) only spent one day in the NCCU. There were no (0%, 0/67) anesthesia-related procedural complications. One (1.5%, 1/67) intra-procedural complication was an aneurysm rupture during attempted coiling. There were five (7.4%, 5/67) post-procedural complications: two (3.0%, 2/67) groin hematomas, two (3.0%, 2/67) permanent neurologic events (left lower extremity hypoesthesia and left upper extremity hemiparesis), and one (1.5%, 1/67) temporary neurologic event (aphasia). Post-procedural complications were associated with longer hospital LOS (p=0.02), but not with longer NCCU LOS. No acute management changes occurred for the five patients that developed post-procedural complications. There were no (0%, 0/67) 30-day readmissions.

Conclusion

The overall incidence of post-procedure complications was low. In the future, a possible viable way to reduce hospital costs may involve utilizing a hospital unit that could closely monitor patients but only for a short period of time post-procedure.

## Introduction

The estimated prevalence of unruptured intracranial aneurysms (UIAs) is 3.2% [1]. In clinical practice, the detection rate of these lesions has increased dramatically due to improved screening methods [2]. This has led to complex and ongoing debates about the optimal treatment modality for cerebral aneurysms [3, 4]. However, clearly, there is a trend toward increased utilization of and spending on endovascular treatment (EVT) for UIAs [5, 6]. This has brought about new challenges for effectively using health care resources. Unfortunately, few studies have examined post-EVT of UIAs clinical decision-making and neurologic intensive care unit (NCCU) admission [7-11].

The purpose of the study was to determine if NCCU admission post elective EVT of UIAs is warranted and whether to prevent complications entirely or hasten their detection. In addition, we sought to identify predictors of complications and determine if these complications were serious enough to warrant acute changes in clinical management or adversely affect patient outcomes. Finally, the aim of this study is to develop future post-EVT of UIA protocols to improve safety and reduce cost.

## Materials and methods

Inclusion criteria

This was a single-center, retrospective chart review that obtained Institutional Review Board (IRB) approval (IRB#7304-B5000343). All consecutive patients from the prospectively maintained institutional neuroendovascular registry were queried between January 1, 2010, and March 31, 2020. Inclusion criteria were age ≥18 years, presence of a UIA, and undergoing EVT. Exclusion criteria were any current intracranial hemorrhage or clinical symptoms severe enough to warrant emergent intervention.

Study variables

Clinical variables were defined as procedure date, age, gender, presence of symptoms pre-procedure, and any previous treatment. Lesion variables were defined as the number of aneurysms treated, largest aneurysm dome diameter, and aneurysm parent vessel origin. Treatment variables were defined as the type of intervention, type of device used, and the number of coils deployed. Post-treatment variables were defined as post-procedure anticoagulation medicine used, the post-procedure anti-platelet medication used, and unit disposition post-treatment. Discharge variables were defined as NCCU length of stay (LOS), hospital LOS, mortality, and whether the patient had 30-day readmission. Outcome measures are defined as anesthesia complications, intra-procedure complications, post-procedure complications, whether an additional intervention was performed, and if the complication warranted a change in the patient’s hospital unit disposition. The clinical, lesion and post-treatment variables were compared to discharge variables. Clinical, lesion, treatment, and discharge variables were compared to outcome measures. Post-treatment variables were compared to post-procedure complications. Outcome measures were compared to unit disposition post-treatment.

Statistical analysis

A sample of convenience was utilized. An a priori power analysis was not performed since consecutive patients were reviewed. Certain variables were dichotomized as either present or absent. If variables were not easily identified after reviewing the electronic medical record, they were considered null and excluded from the analysis. Independence was assumed between distinct procedures performed on the same patient. Descriptive analyses are reported as mean (range, ± 1 SD). Pearson’s correlation coefficients were estimated for all comparisons, and significance was assessed using Monte Carlo simulation of Fisher’s exact tests. A p-value of ≤0.05 was considered statistically significant. Statistical analyses were performed using SAS (version 9.4; SAS Institute Inc).

## Results

Clinical variables

A total of 4,498 charts were screened dating back to January 1, 2010; however, the first UIA to meet inclusion criteria was treated on April 7, 2016. As a result, a total of 67 procedures met inclusion criteria that were performed on 58 patients (Table 1).

**Table 1:** Study sample of elective endovascular treatment of unruptured intracranial aneurysms. Mean age (range, ± 1 SD).

Variable		Value
Total procedures		67
Initial		56
Retreatment		9
Patients		58
Female		54
Male		4
Age (years)		59.3 (22-84, ±15.1)
Symptomatic		
No		59
Yes		8

**Figure 1:**
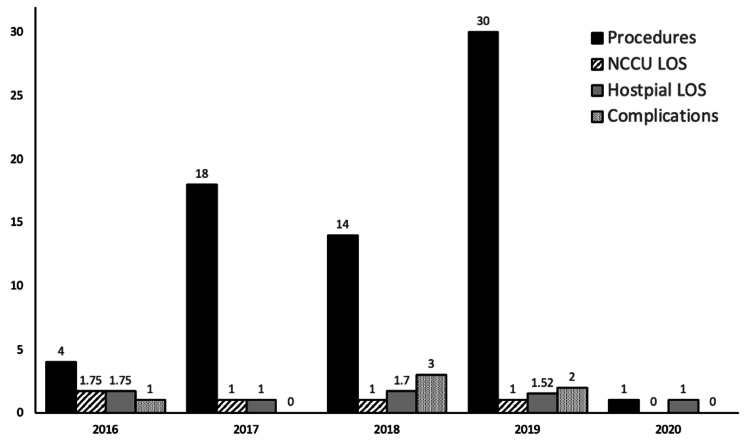
Number of procedures, length of stay, and complication incidence over time. Mean hospital and NCCU length of stay reported in days. The first and last procedures to meet inclusion criteria were performed on April 7, 2016 and March 11, 2020, respectively. NCCU: Neurologic intensive care unit; LOS: Length of stay.

There was a trend towards increased utilization of EVT for UIA during the sample time period, with 44.8% (30/67) of cases being performed in 2019 (Figure 1).

Most patients (93.1%, 54/58) were female, and the mean age was 59.3 (22-84, ±15.1). In addition, most patients were asymptomatic (88.0%, 59/67) and had never undergone a previous EVT for their UIA (83.6% 56/67). Symptomatic UIA was positively correlated with increased NCCU LOS (r=0.48). However, this relationship was not significant (p=0.10). No associations were found with the age, gender, and previous EVT for NCCU LOS and hospital LOS. There were no associations with procedure date, age, gender, whether symptomatic, or previous EVT for post-procedure complications.

Lesion variables

Typically only one UIA was treated (94%, 63/67) during a single procedure, although there were instances of multiple UIAs being treated (two UIAs: 4.5%, 3/67; three UIAs: 1.5%, 1/67) (Table 2).

**Table 2:** Aneurysms treated, location, and morphology. Mean dome diameter (±1 SD). ACA: Anterior cerebral artery; ACOM: Anterior communicating artery; ICA: Internal cerebral artery; MCA: Middle cerebral artery; OphA: Ophthalmic artery; PCA: Posterior cerebral artery; PCOM: Posterior communicating artery; PICA: Posterior inferior cerebellar artery.

Variable		Frequency	Diameter
Aneurysms treated		67	6.6 ± 3.9
1		63	
2		3	
3		1	
Aneurysm Location			
ACA		2	3
ACOM		9	7.2
Basilar		9	5.3
ICA		29	7.4
MCA		7	5.9
OphA		3	5.8
PCA		2	9
PCOM		5	6.7
PICA		1	5.5

The mean dome diameter for all UIA was 6.6 mm (2-20, ±3.9), and most were in the anterior circulation (76.1%, 51/67). The most common parent vessels were the internal carotid artery (ICA) (43.2%, 29/67), the anterior communicating artery (ACOM) (13.4%, 9/67), and the basilar artery (13.4%, 9/67). There were no associations found between the number of UIA treated and NCCU or hospital LOS. Increasing UIA diameter was associated with longer NCCU LOS (p=0.03) but not hospital LOS. ACOM UIA was correlated with longer NCCU LOS (r=0.38); however, this relationship was not significant (p=0.15); ACOM UIA had significantly longer hospital LOS (p=0.04). There was a correlation between the increasing number of UIA treated and the incidence of post-procedure complications (r=0.29). However, this relationship was not significant (p=0.09). There was no association between UIA diameter and post-procedural complications. ACOM UIA were more likely to develop post-procedural complications (p=0.02).

Treatment variables

**Figure 2:**
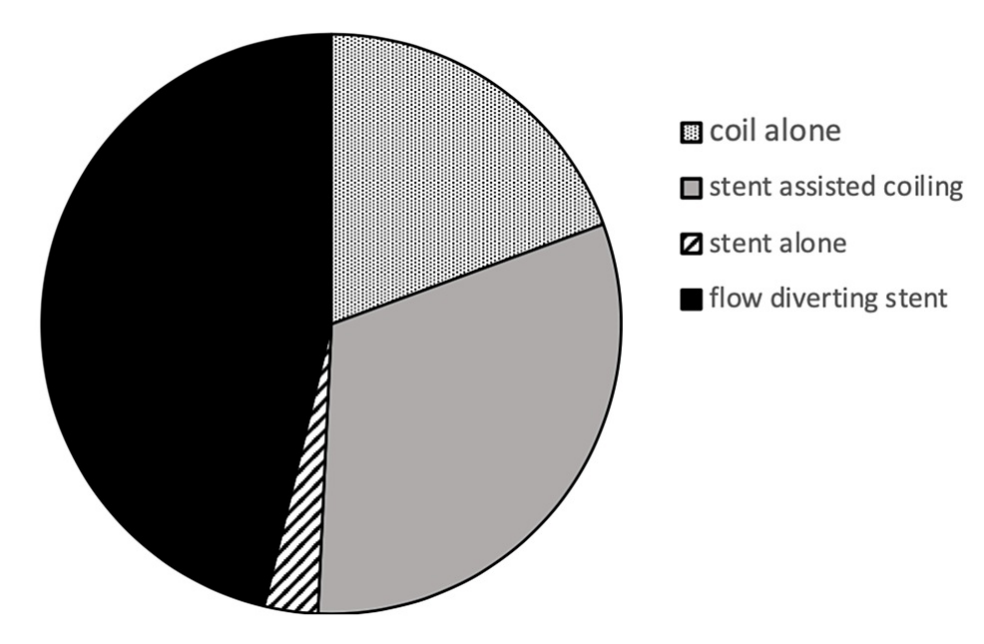
Type of device used to treat unruptured intracranial aneurysm. Sole flow diverting stents were used for 46.2% (31/67), stent-assisted coiling was used for 31.3% (21/67), sole coil-based intervention was used for 19.4% (13/67), and stent alone was used for 3.0% (2/67).

Sole flow diverting stents (FDS) were the most common device used (46.2%, 31/67), followed by stent-assisted coiling (31.3%, 21/67), and then sole coil-based intervention (19.4%, 13/67) (Figure 2).

When deployed, the mean number of coils used was 4.3 (1-18, ±3.14). Utilization of FDS was correlated with shorter NCCU LOS (r=-0.32); however, this relationship was not significant (p=0.20). There were no associations found with the specific model device used or the number of coils deployed with NCCU LOS or hospital LOS. There were no associations between the type of device used and the number of coils deployed with post-procedural complications.

Post-treatment variables

Only one patient (1.5%, 1/67) was placed on immediate post-procedure therapeutic anticoagulation in the form of a heparin drip. Most patients were placed on immediate post-procedure dual anti-platelet medications (aspirin and clopidogrel: 79.1%, 53/67; aspirin and ticagrelor: 3.0%, 2/67), while others received a single antiplatelet agent (aspirin: 6.0%, 4/67; clopidogrel: 3.0%, 2/67). For post-treatment disposition, 55.2% (37/67) went to non-NCCU hospital floor units, 43.2% (29/67) went to the NCCU, and 1.5% (1/67) were not admitted and discharged directly home. Utilization of no antiplatelet medications and aspirin alone was correlated with a longer and shorter NCCU LOS (r=0.56, r=-0.38), respectively. However, these relationships were not significant (both p>0.07). There were no other associations between antiplatelet medications and NCCU LOS or hospital LOS. There were no associations between antiplatelet medication usage and post-treatment disposition with post-procedural complications.

Discharge variables

The total mean NCCU LOS was 1.07 days (range 1-4, ±0.5), and 96.6% (28/29) only spent one day in the NCCU. The total mean hospital LOS was 1.43 days (1-9, ±1.5). There was one mortality (1.5%, 1/67) due to an intra-procedure aneurysm rupture. There were no (0%, 0/67) 30-day readmissions. Post-procedural complications were associated with a longer hospital LOS (p=0.02), but not with a longer NCCU LOS.

Outcome measures

There were no (0%, 0/67) anesthesia-related procedural complications. One (1.5%, 1/67) intra-procedural complication was a 20 mm ACOM aneurysm rupture during a coiling attempt. An external ventricular drain (EVD) was immediately placed; however, the patient ultimately died during the subsequent NCCU stay. There were a total of five (7.4%, 5/67) post-procedural complications: two (3.0%, 2/67) groin hematomas due to pseudoaneurysms, two (3.0%, 2/67) permanent neurologic events (left lower extremity hypoesthesia and left upper extremity hemiparesis), and one (1.5%, 1/67) temporary neurologic event (aphasia) (Table 3).

**Table 3:** Complications of endovascular treatment of unruptured intracranial aneurysms. Age reported in years; Number of aneurysms treated in one procedure; Largest aneurysm dome diameter; Parent vessel aneurysm location; Number of coils used; Length of stay reported in days. ACOM: Anterior communicating artery; EVD: External ventricular drain; F: Female; FDS: Flow diverting stent; ICA: Internal cerebral artery; LOS: Length of stay; N: No; NCCU: Neurologic intensive care unit; Y: Yes; -: Data not available.

Patient	Symptomatic	Previous Treatment	Aneurysms , Diameter, Vessel	Treatment Type	Post-procedure Medication	Disposition	NCCU, Hospital LOS	Complication	Management Change	Outcome
57, F	Yes, headache	N	1, 20 mm, ACOM	Coils (6)	None	NCCU	4, 4	Intra-procedure rupture	EVD	Mortality
84, F	N	N	1, 5.5 mm, ACOM	Stent, Coils (3)	Aspirin, Clopidogrel	Floor	0, 1	Groin hematoma	None	Resolved
69, F	N	N	3, 5 mm, ICA	FDS	Aspirin, Clopidogrel	Floor	0, 3	Groin hematoma	None	Resolved
58, F	N	N	1, 6 mm, ACOM	Stent, Coils (4)	Aspirin, Clopidogrel	NCCU	1, 2	Mono hypoesthesia	None	Permanent
46, F	N	Y	1, -, ACOM	Stent, Coils (-)	Aspirin, Clopidogrel	NCCU	1, 9	Mono hemiparesis	None	Permanent
68, F	N	N	1, 7 mm, ACOM	FDS	Aspirin, Clopidogrel	Floor	0, 2	Aphasia	None	Resolved

Neither any additional interventions were performed, nor did the patient’s hospital unit disposition change for the five patients that developed post-procedural complications.

## Discussion

Opting for EVT of UIAs involves weighing the risk of immediate procedure-related complications against the risk of future rupture; the latter is supported by robust clinical data [12, 13]. Results from the International Study of Unruptured Intracranial Aneurysms (ISUIA) trial and PHASES score have shown that not all UIA need to be immediately treated [12, 13]. In our study, the mean age was 59.3 years old, the mean dome diameter was 6.6 mm, and 52.2% (35/67) of UIA were in the middle cerebral artery (MCA), anterior cerebral artery (ACA), posterior communicating artery (PCOM), or posterior circulation vessels. While 16.4% (11/67) of the UIA treated had undergone the previous EVT, we did not record if there was previous subarachnoid hemorrhage (SAH). We also did not record the patient nationality or presence of hypertension. Opting for EVT of UIA may also be dictated by patient preference. Ultimately, we did not record the specific indication for EVT for a particular UIA.

An estimated 55% of all patients admitted to the NCCU are for monitoring alone. As many as 78% of these monitoring alone patients, as defined by their Acute Physiology, Age, Chronic Health Evaluation (APACHE) III score, are at low risk of receiving subsequent active life-supporting treatment [14]. Therefore it is crucial to identify which patients derive benefit from or absolutely require the level of monitoring and intervention that only an NCCU can provide. Critical care units are substantially more expensive than other hospital units, and their direct costs are rising [15, 16]. Complications of EVT of UIAs are known but rare, making their indirect costs challenging to measure [17-22]. Hospital charges for EVT of UIAs often exceed those for surgical clipping [17]. However, Medicare reimbursement payments for aneurysm treatment are substantially lower than hospital costs [23]. This disparity, coupled with the increased utilization of EVT, presents a tremendous opportunity to improve post-procedural EVT of UIA care.

In 2008, the results of the Analysis of Treatment by Endovascular Approach of Nonruptured Aneurysms (ATENA) study were published [19]. In this landmark prospective international multicenter study of 649 patients harboring 1100 aneurysms undergoing elective EVT of UIA, the overall complication rate was 15.4% [19]. Since endovascular techniques and devices have evolved, the 30-day morbidity and mortality rates were 1.7% and 1.4%, respectively [19]. Contemporary meta-analyses have reported overall complication rates of 3.69-4.96% and 0.57% mortality rates, which aligns with our experience reported herein [17, 18, 20]. Previously identified predictors of complications include female gender, diabetes, hyperlipidemia, pre-existing cardiac comorbidities, wide aneurysm neck >4 mm or a dome:neck ratio >1.5, posterior circulation aneurysms, stent-assisted coiling, and stenting alone [18].

Identifying the most likely precise timing of post-procedure complications may represent an opportunity to reduce morbidity and mortality. For example, Arias EJ et al. looked at 687 elective intracranial aneurysm coilings and found 74% of complications occurred within 4 hours of the procedure, 14.8% occurred within 4-12 hours, 3.7% happened within 12-24 hours, and 7.4% of complications occurred >24 hours after intervention [21]. Half of the complications that occurred <4 hours after intervention required treatment or resulted in permanent deficits, whereas those that occurred >12 hours after intervention were discharged home without deficits [21]. Furthermore, others have reported that 88% of complications occur within 24 hours of intervention [22]. In our study, the mean NCCU LOS was 1.07 days, and the admission to NCCU likely would not have hastened the detection of, reduced the morbidity associated with, or prevented the post-procedure complications.

Others have retrospectively examined elective EVT of UIA and proposed safe, cost-saving solutions [7-11] (Table 4).

**Table 4:** Previous publications on post-procedure cost savings initiatives. EVT: Endovascular treatment; LOS: Length of stay; PACU: Post-anesthesia care unit; UIA: Unruptured intracranial aneurysm.

Article	Level of Evidence	Design	Study Sample	Measures	Results
Stetler WR et al. (2017) [7]	III	Retrospective, single-center	Elective coiling UIA, n=311	Predictors post-procedure complications, cost ICU vs. stepdown vs. telemetry unit	6.4% complication rate, cost savings 57% stepdown and 32% telemetry compared to ICU
Zakhari N et al. (2016) [8]	III	Retrospective, single-center	Elective coiling UIA, n=107	30-day adverse events, dichotomized to early <2 day LOS discharge to late >2 day LOS discharge	15.47% adverse events, no differences in early vs. late discharge
Eisen SH et al. (2015) [9]	III	Retrospective, single-center	Elective EVT UIA, n=170	96-hour adverse events, disposition ICU vs. PACU	9.1% adverse events, incidence of permanent deficits or mortality same for ICU vs. PACU
Zanaty M et al. (2016) [10]	III	Retrospective, single-center	Elective pipeline UIA, n=130	Protocol adherence: planned discharge home 6 hours post-procedure, overall incidence of complications	90.6% patients discharged home within 6 hours post-procedure, 0.75% overall complication rate
Burrows AM et al. (2013) [11]	III	Retrospective, single-center	Elective EVT UIA, n=200	Post-procedure complications ICU vs. floor, change in acute management post-procedure, LOS	Complication rate same for ICU vs. floor, 0.8% change in acute management post-procedure, ICU longer LOS

Based on their experience, many of these authors have concluded that routine post-EVT of UIA does not warrant automatic NCCU admission post-procedure [9-11]. However, we are unaware of any high-quality, prospective randomized studies comparing different hospital unit dispositions and outcomes. The principles of postoperative care include early identification and management of potential complications to reduce morbidity. At our institution, an anesthesiology team and neurointerventional proceduralist make a joint decision after treatment for patient disposition. Typically patients go to a post-anesthesia care unit (PACU) with 1:1 nursing to patient staffing for a period of 1-4 hours before going to a different hospital unit. There are several limitations to this study. First, this was a single-center retrospective study. Our sample size is also smaller as compared to other previously published papers. Second, we did not record the duration and type of anesthesia used, although no intra-procedure anesthesia complications occurred. Next, it is difficult to generalize the findings of this paper and others to facilities with varying staff and facility resources. Fourth, we only looked at electively treated UIA, and our results should not be generalized to ruptured aneurysms. Lastly, neuroendovascular treatment is an evolving field, which makes it challenging to generalize findings across rapidly changing techniques and devices.

## Conclusions

In this study, we sought to scrutinize routine NCCU admission post-EVT of UIAs in regards to the incidence and treatment of procedural complications. The overall incidence of morbidity and mortality was low, and the presence of complications often did not change clinical management. Literature shows that predictors of complications are readily identifiable pre-intervention. Some patients could be delegated as low risk in the absence of intra-procedure complications such as hemorrhage or thrombosis. A possible, viable way to reduce hospital costs may involve utilizing a hospital unit that could closely monitor patients but only for a short period of time post-procedure. However, any protocol should be driven by patient safety and outcomes.

## References

[1] Vlak MH, Algra A, Brandenburg R, Rinkel GJ (2011). Prevalence of unruptured intracranial aneurysms, with emphasis on sex, age, comorbidity, country, and time period: a systematic review and meta-analysis. Lancet Neurol.

[2] Sharma M, Ugiliweneza B, Fortuny EM (2019). National trends in cerebral bypass for unruptured intracranial aneurysms: a National (Nationwide) Inpatient Sample analysis of 1998-2015. Neurosurg Focus.

[3] Molyneux AJ, Birks J, Clarke A, Sneade M, Kerr RS (2015). The durability of endovascular coiling versus neurosurgical clipping of ruptured cerebral aneurysms: 18 year follow-up of the UK cohort of the International Subarachnoid Aneurysm Trial (ISAT). Lancet.

[4] Spetzler RF, McDougall CG, Zabramski JM (2015). The barrow ruptured aneurysm trial: 6-year results. J Neurosurg.

[5] Huang MC, Baaj AA, Downes K, Youssef AS, Sauvageau E, van Loveren HR, Agazzi S (2011). Paradoxical trends in the management of unruptured cerebral aneurysms in the United States: analysis of nationwide database over a 10-year period. Stroke.

[6] Brinjikji W, Rabinstein AA, Nasr DM, Lanzino G, Kallmes DF, Cloft HJ (2011). Better outcomes with treatment by coiling relative to clipping of unruptured intracranial aneurysms in the United States, 2001-2008. AJNR Am J Neuroradiol.

[7] Stetler WR Jr, Griauzde J, Saadeh Y (2017). Is intensive care monitoring necessary after coil embolization of unruptured intracranial aneurysms?. J Neurointerv Surg.

[8] Zakhari N, Lum C, Quateen A, Iancu D, Lesiuk H (2016). Next day discharge after elective intracranial aneurysm coiling: is it safe?. J Neurointerv Surg.

[9] Eisen SH, Hindman BJ, Bayman EO, Dexter F, Hasan DM (2015). Elective endovascular treatment of unruptured intracranial aneurysms: a management case series of patient outcomes after institutional change to admit patients principally to postanesthesia care unit rather than to intensive care. Anesth Analg.

[10] Zanaty M, Daou B, Chalouhi N (2016). Same-day discharge after treatment with the pipeline embolization device using monitored anesthesia care. World Neurosurg.

[11] Burrows AM, Rabinstein AA, Cloft HJ, Kallmes DF, Lanzino G (2013). Are routine intensive care admissions needed after endovascular treatment of unruptured aneurysms?. AJNR Am J Neuroradiol.

[12] Wiebers DO, International Study of Unruptured Intracranial Aneurysms Investigators (2003). Unruptured intracranial aneurysms: natural history, clinical outcome, and risks of surgical and endovascular treatment. Lancet.

[13] Greving JP, Wermer MJ, Brown RD (2014). Development of the PHASES score for prediction of risk of rupture of intracranial aneurysms: a pooled analysis of six prospective cohort studies. Lancet Neurol.

[14] Zimmerman JE, Junker CD, Becker RB, Draper EA, Wagner DP, Knaus WA (1998). Neurological intensive care admissions: identifying candidates for intermediate care and the services they receive. Neurosurgery.

[15] Milbrandt EB, Kersten A, Rahim MT (2008). Growth of intensive care unit resource use and its estimated cost in Medicare. Crit Care Med.

[16] Halpern NA, Pastores SM (2010). Critical care medicine in the United States 2000-2005: an analysis of bed numbers, occupancy rates, payer mix, and costs. Crit Care Med.

[17] Alshekhlee A, Mehta S, Edgell RC (2010). Hospital mortality and complications of electively clipped or coiled unruptured intracranial aneurysm. Stroke.

[18] Algra AM, Lindgren A, Vergouwen MD, Greving JP, van der Schaaf IC, van Doormaal TP, Rinkel GJ (2019). Procedural clinical complications, case-fatality risks, and risk factors in endovascular and neurosurgical treatment of unruptured intracranial aneurysms: a systematic review and meta-analysis. JAMA Neurol.

[19] Pierot L, Spelle L, Vitry F (2008). Immediate clinical outcome of patients harboring unruptured intracranial aneurysms treated by endovascular approach: results of the ATENA study. Stroke.

[20] Kang XK, Guo SF, Lei Y, Wei W, Liu HX, Huang LL, Jiang QL (2020). Endovascular coiling versus surgical clipping for the treatment of unruptured cerebral aneurysms: direct comparison of procedure-related complications. Medicine (Baltimore).

[21] Arias EJ, Patel B, Cross DT 3rd, Moran CJ, Dacey RG Jr, Zipfel GJ, Derdeyn CP (2014). Timing and nature of in-house postoperative events following uncomplicated elective endovascular aneurysm treatment. J Neurosurg.

[22] Kameda-Smith MM, Klurfan P, van Adel BA, Larrazabal R, Farrokhyar F, Bennardo M, Gunnarsson T (2018). Timing of complications during and after elective endovascular intracranial aneurysm coiling. J Neurointerv Surg.

[23] Brinjikji W, Kallmes DF, Lanzino G, Cloft HJ (2012). Hospitalization costs for endovascular and surgical treatment of ruptured aneurysms in the United States are substantially higher than Medicare payments. AJNR Am J Neuroradiol.

